# Halogenated Volatile Organic Compounds in Water Samples and Inorganic Elements Levels in Ores for Characterizing a High Anthropogenic Polluted Area in the Northern Latium Region (Italy)

**DOI:** 10.3390/ijerph18041628

**Published:** 2021-02-08

**Authors:** Mario Vincenzo Russo, Ivan Notardonato, Alberto Rosada, Giuseppe Ianiri, Pasquale Avino

**Affiliations:** 1 Department of Agriculture, Environment and Food Sciences (DiAAA), University of Molise, 86100 Campobasso, Italy; ivan.notardonato@unimol.it (I.N.); g.ianiri@studenti.unimol.it (G.I.); 2 ENEA Cassacia Research Center, 00060 Rome, Italy; alberto.rosada@enea.it

**Keywords:** anthropogenic sources, natural sources, halogen, element, analysis, arsenic, pollution, public health, water, soil

## Abstract

This paper shows a characterization of the organic and inorganic fraction of river waters (Tiber and Marta) and ores/soil samples collected in the Northern Latium region of Italy for evaluating the anthropogenic/natural source contribution to the environmental pollution of this area. For organic compounds, organochloride volatile compounds in Tiber and Marta rivers were analyzed by two different clean-up methods (i.e., liquid–liquid extraction and static headspace) followed by gas chromatography–electron capture detector (GC-ECD) analysis. The results show very high concentrations of bromoform (up to 1.82 and 3.2 µg L^−1^ in Tiber and Marta rivers, respectively), due to the presence of greenhouse crops, and of chloroform and tetrachloroethene, due to the presence of handicrafts installations. For the qualitative and quantitative assessment of the inorganic fraction, it is highlighted the use of a nuclear analytical method, instrumental neutron activation analysis, which allows having more information as possible from the sample without performing any chemical-physical pretreatment. The results have evidenced high levels of mercury (mean value 88.6 µg g^−1^), antimony (77.7 µg g^−1^), strontium (12,039 µg g^−1^) and zinc (103 µg g^−1^), whereas rare earth elements show levels similar to the literature data. Particular consideration is drawn for arsenic (414 µg g^−1^): the levels found in this paper (ranging between 1 and 5100 µg g^−1^) explain the high content of such element (as arsenates) in the aquifer, a big issue in this area.

## 1. Introduction

The current legislation defines pollution as any modification of the atmospheric air, due to the introduction into it of one or more substances in quantities and with characteristics such as to damage or constitute a danger to human health or to the quality of the environment, or such as to damage material assets or compromise legitimate uses of the environment [1,2]. Anthropogenic and/or natural sources are recognized worldwide as responsible for various and dangerous episodes of pollution. Natural sources of pollution have always existed in nature (volcanic eruptions, geothermal activities, spontaneous fires, high wind events, atmospheric resuspension and transport of natural particulate matter from arid regions), which alter the composition and chemical–physical characteristics of the atmosphere [3,4,5,6,7,8]. On the other hand, many of the pollutants generated by anthropogenic sources are the same as those produced by natural events, but the morphological/orographic characteristics of each location, together with the meteorological factors, favor their accumulation, sometimes reaching high levels of concentration and triggering the formation of further pollutants through transformations chemical. The effects of pollutants on different organisms vary according to the concentration in the air, the residence time and their physical-chemical characteristics.

Nowadays, environmental contamination episodes are becoming very frequent. From an environmental point of view, a chemical characterization is really important because it helps the evaluation of the environmental impact of anthropogenic activities on urban areas and industrial and agricultural sites [9]. The analytical issue is complicated due to a great difference between estimated emissions and calculated depositions. All these considerations are related to definitions of critical loads and critical levels, issued by the World Health Organization (WHO) [1]. Critical load is defined as the quantitative assessment of exposure to one or more pollutants below which, to the current state of the art, do not have significant adverse effects; the critical level is the pollutant concentration into the atmosphere above which, according to the current state of knowledge, there may be direct harmful effects for recipients such as human beings, plants, ecosystems [9,10].

Among thousands of organic compounds present in environmental matrices, halocarbons, i.e., organic chemical molecules containing at least a chemical covalent bond between a carbon atom and one (or more) halogen atoms [11,12], are very toxic (and some of them also carcinogenic), the most common are fluorine, chlorine, bromine and iodine [13,14]. Every environmental compartment, i.e., atmosphere, water and soil, suffers from contamination of halocarbon compounds, which come from anthropogenic sources such as refrigerants (release into the atmosphere), industrial emissions (accidental solvent release into the environment) and slash-and-burn agriculture.

On the other hand, the inorganic fraction essentially regards heavy metals. This is a general term for identifying a group of metals and metalloids with an atomic density greater than 5 g cm^−3^ [15]. The human activity represented by industries, by urban settlements (intended in particular as discharges of wastewater and purification plants of the same), by mining and agricultural activities, involve a release (in the air, in the soil and in the waters) of considerable quantities of metals (in particular Cd, Zn, Pb, and Hg), which pass directly, by leaching from the soils or linked to sediments, in water basins. Here they can undergo biological and chemical transformations, which lead to an accumulation in the environment (in the form of sediments) and in organisms, both plant and animal, thus carrying out their polluting action [16,17]. Although the current exposure to anthropogenic sources is of prevailing toxicological importance, that of natural sources has been fundamental for the development, in living organisms, of detoxification mechanisms aimed at the elimination and reduction, if not abatement, the dangerousness of metals. These mechanisms allow some animal species to withstand high tissue concentrations, which, conversely, can be toxic to others, without suffering any damage [18]. Their critical load can be defined as “a quantitative estimate of an exposure to one or more elements below which no significant harmful effects on specific sensitive elements of the environment occur according to current knowledge”. Even if critical loads for some heavy metals such as cadmium, lead, copper, nickel and zinc [9] were determined, many metals still show great discrepancies between emission estimates and measured deposition.

Starting with these considerations, the authors would like to explore how anthropogenic activities and natural sources can affect an area located in the Northern Latium, Central Italy. Organic compounds such as halocarbons and the inorganic fraction in terms of elements were determined: the first ones can be considered an index of the anthropogenic sources, whereas the second ones can be considered an index of natural sources, i.e., from the continental crust.

Ore and river-waters from Tiber and Marta river samples were collected in different intensive sampling campaigns aimed at identifying the anthropogenic/natural impact of different activities in this area. Organohalogen compounds were determined by a chromatographic method, whereas the inorganic elements by means of nuclear analytical technique such as Instrumental Neutron Activation Analysis (INAA) [19], i.e., a nuclear methodology which allows detecting ultra-trace levels avoiding any physical-chemical pretreatment [20,21]. To the authors’ knowledge, this is the deepest study of this area, which is very important from geological and sanitary points of view for two essential reasons: (i) the (so-far believed) levels of U and Th have supposed this area to be a uranium province and (ii) the highest concentrations of As, found annually in potable waters of the Viterbo area during the hot periods, have always forced the public health authorities to forbid the use of potable water for domestic uses with serious difficulties and damages for the population. It should be underlined that Italy is currently under European infringement proceeding for this last issue.

## 2. Materials and Methods

### 2.1. Sampling Site

**Figure 1:**
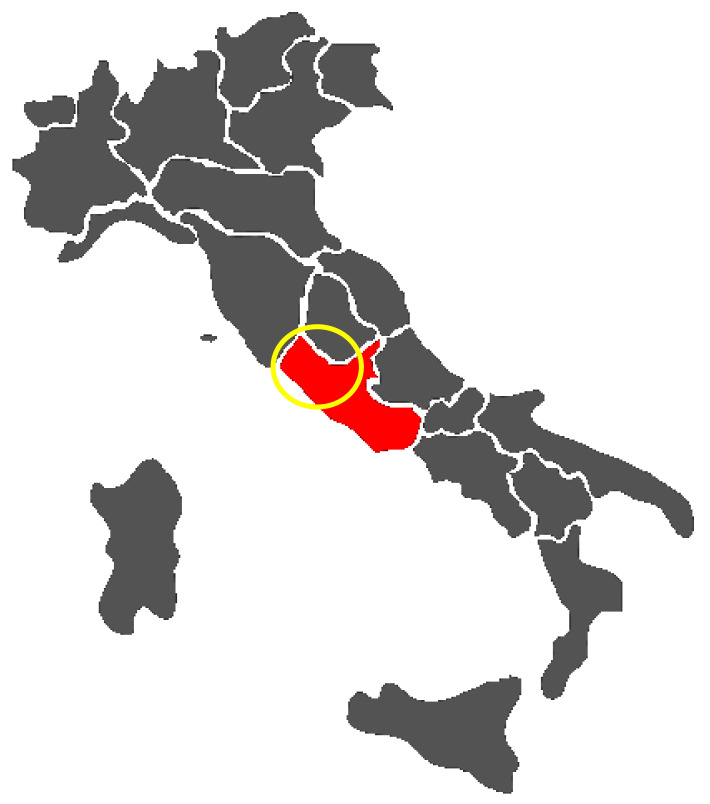
Map of the Latium region with the area investigated in this study.

Soil and river water samples were sampled across the Northern Latium, throughout the volcanic area between Tuscany and the sea (Tyrrhenian Sea), in an area of 6363 km^2^ (472,533 inhabitants). The water was sampled annually in the period April 2015–March 2016: particularly, 48 water samples were taken in spring-, summer- and fall-time near Viterbo (42°25′07″ N 12°06′15″ E) and Tarquinia (42°14′57″ N 11°45′22″ E) and at the mouths of the Tiber and Marta River (42°31′59.89″ N 11°55′51.49″ E), and in the Bracciano area (42°06′ N 12°11′ E). On the other hand, 110 ores were collected from 8 different areas in the North Latium region between July and August 2015: Bracciano area (42°06′ N 12°11′ E), Ceriti Mt. (42°09′03.02″ N 11°54′34.31″ E), Fate Mt. (41°24′17.89″ N 13°18′29.52″ E), Sabatini Mt. (42°10′ N 12°15′ E), Oriolo Romano (42°09′28.08″ N 12°08′16.08″ E), Solfatara basin (42°25′17″ N 11°52′19″ E), Vulsini Mt. (42°40′ N 11°55′ E), Acqua Rossa basin (42°29′ N 12°08′ E). Figure 1 shows the sampling sites located across Northern Latium: in particular, it can be evidenced that the sampling was performed through the volcanic area between Tuscany and the sea (Tyrrhenian Sea).

### 2.2. Organic Fraction

Chloroform (CHCl_3_), trichloroethane (CHCl_2_CH_2_Cl), hexachloroethane (CCl_3_CCl_3_), tetrachloromethane (CCl_4_), chlorobenzene (C_6_H_5_Cl), trichloroethene (CCl_2_CHCl), dichlorobromomethane (CHBrCl_2_), bromoform (CHBr_3_), tetrachloroethene (CCl_2_CCl_2_), dibromochloromethane (CHClBr_2_) and tetrachloroethane (CHCl_2_CHCl_2_) (analytical reagent grade, purity ≥ 99.5%), methanol (pesticide grade), and NaCl (RPE) were all from Carlo Erba (Milan, Italy). Solutions of each halocarbon standard were prepared at the following concentrations: CHCl_3_—7.4 g L^−1^; CCl_3_CCl_3_—6.70 g L^−1^; CCl_4_—0.8 g L^−1^; CCl_2_CHCl—7.3 g L^−1^; CHBrCl_2_—9.9 g L^−1^; CHCl_2_CH_2_Cl—8.6 g L^−1^; CCl_2_CCl_2_—9.7 g L^−1^; CHClBr_2_—12.2 g L^−1^; C_6_H_5_Cl—5.2 g L^−1^; CHBr_3_—14.5 g L^−1^; CHCl_2_CHCl_2_—9.6 g L^−1^. From these solutions, a single mixed solution containing all the compounds was prepared. The analysis of hexane extracts was performed by the breaking method (analysis of the standard, analysis of the samples, analysis of the standard). Water samples (5 L each sample) were collected at a depth of approximately 50 cm below the surface and stored in polypropylene bottles at temperatures below 4 °C.

A Dani gas chromatograph, mod. 86.10 HT (Dani, Monza, Italy), equipped with a Vocol capillary column “wide-bore”, 60 m × 0.75 µm × 1.5 µm film thickness (Supelco) and a programmed temperature vaporizing (PTV) injector and an electron capture detector (ECD) connected with a Shimadzu CR 3A data processor, was used for sample analysis. The PTV was set up at 50 °C for 1 min, and then increased to 240 °C at 800 °C min^−1^; it operates with total sample injection. The detector temperature was at 250 °C. The column temperature was kept at 40 °C for 8 min: after, it was increased to 900 °C at 4 °C min^−1^. Nitrogen (UPP) was used as a carrier gas and as auxiliary gas (make-up) at 15 and 35 mL min^−1^, respectively.

This laboratory developed different extraction procedures [22,23,24,25,26] for analyzing organic compounds in different environmental and clinical matrices [27,28,29,30,31,32,33]. In this case, two different extractions were performed, i.e., liquid–liquid extraction (LL) and headspace analysis (HS). Basically, the headspace analysis is simpler than the LL method and requires poor sample manipulations. The liquid–liquid extraction was carried out on the water sample using a round flask (0.5 L) in which 0.5 L of water with NaCl (10 g L^−1^) were extracted with 500 µL of n-hexane. After 15 minutes of vigorous shaking using a magnetic stirrer and after separation, the organic extract was recovered and injected into the GC for analysis. For headspace analysis, a river water sample (110 mL) was placed in a vial, and NaCl (10 g L^−1^) was added. The vial was then closed with a cap containing polytetrafluoroethylene (PTFE)-coated rubber septum and kept at 50 °C for 60 min. Headspace gas-chromatographic analysis was performed by manually injecting 1.0 mL of the vial headspace from each sample by means of a 1.5-mL Hamilton gas-tight syringe (temperature kept at 50 °C).

**Figure 2:**
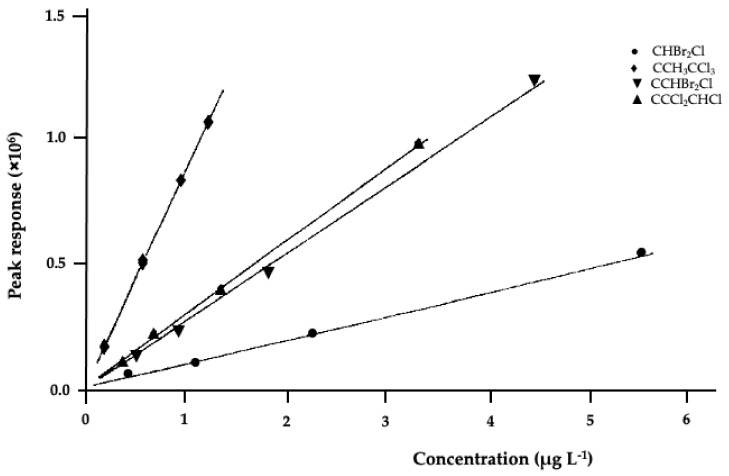
Calibration curves of CH_3_CCl_3_, CCl_2_CHCl, CHBrCl_2_ and CHBr_2_Cl.

Figure 2 shows the calibration curves of 1,1,1-trichloroethane, trichloroethylene, dichlorobromomethane and dibromochloromethane, considered representative of these compounds: a good response in the linearity range investigated is evidenced.

Table 1 reports all the analytical data for both the procedures used in this study, i.e., static headspace analysis and liquid–liquid extraction followed by GC-ECD analysis.

All the values reported in tables are the average among five determinations, and the relative standard deviations (RSDs) are in the RSD range for a standard solution analyzed by both methods.

### 2.3. Inorganic Fraction

The inorganic element evaluation was carried out using a highly specific nuclear method, i.e., INAA, which involves collecting as much information as possible without subjecting the sample to any chemical treatment [19,21,34]. For ores/soil, a stratified systematic sampling procedure was applied. The ore sample collection was a superficial systematic sampling by means of geological pick; it interested 30 cm-thick surface layer, at least. Two kg of material for each sample were collected, temporally stored in a plastic bag, and in the lab rapidly underwent the analytical procedure. After sampling, each ore was undergone crushing, homogenization, drying in an oven at 105 °C for removing humidity and storage in the dryer the following. The neutron irradiation was performed in the TRIGA Mark II nuclear reactor at the R.C. Casaccia-ENEA. About 0.5 g of each sample was irradiated in the rotatory rack, Lazy Susan, at 2.6 × 10^12^ n×cm^2^×s^−1^ for 12 h. Table 2 shows the radioisotopes used to calculate the concentration of the analyzed elements along with the nuclear data and the relative gamma-spectrometry interferences [35,36,37].

The gamma spectrometry measurements were performed by means of an HPGe detector (EG&G Ortec, Oak Ridge, Tennessee) with an energy resolution (FWHM) of 1.75 keV 1332.5 keV, a peak/Compton ratio ^60^Co 55.1 and a relative efficiency of 22% at 1332.5 keV. The measurements were performed and analyzed using the Silena Silgamma software produced by Silena (Milan, Italy). According to the half-life of each element [40,41], more than 40 elements were investigated.

Primary (1 mg mL^−1^ of each one) and standard reference materials (SRMs; five geochemical SRMs such as USGS GRX-1, GRX-3, GRX-4, GRX-5, GRX-6) [42,43] were used to assess the quality control.

## 3. Results

It should be noted that this study is appropriate and relevant for evaluating the environmental contamination in specific areas with high anthropogenic pollution. The exportability of such investigation, including the analytical methods used for achieving the results, is based on the study of the organic fraction (i.e., halocarbons) and an inorganic fraction (i.e., heavy metals) with the aim to find out the main representative compounds/metals responsible of possible environmental damages.

### 3.1. Organic Fraction

The determination of the organic fraction was addressed to the evaluation of halocarbons. Before performing the determinations, the analytical methodology to be used was deeply studied. This step is fundamental for the knowledge about the reliability of the data obtained. First, all the analytical parameters in terms of linearity range, limit of detection (LOD), repeatability and recoveries were evaluated. Looking at the data (Table 1), it can be noted that the linearity response is good in the studied concentration ranges and the repeatability (expressed as RSD) is very good (RSD decreases with the increasing concentrations) as well as the recoveries are almost similar about the two methods whereas the LODs are different: for each compound L-L method shows LOD lower that one found for HS analysis. The LODs were determined according to Knoll’s definition [44] (ratio signal and noise, S/N, 3/1). Hence, even if the approach based on HS-GC-ECD is more rapid than the other, the method based on the liquid extraction followed by GC-ECD is more robust and allows to find out such compounds at very low levels. In any case, taking into account that the low limit is 10 µg L^−1^ as the sum of organohalogen compounds [45], both methods are reliable and adequate for estimating these compounds in river water samples. NaCl plays an important role: its presence increases the solute volatility (“salt–effect”) and simultaneously reduces the solubility of such compounds in water [46]. Hence, using both methods, i.e., headspace analysis and/or liquid–liquid extraction followed by GC-ECD, different river water samples collected from Tiber and Marta Rivers were analyzed. The sampling occurred at different points as well as seasons and years.

**Figure 3:**
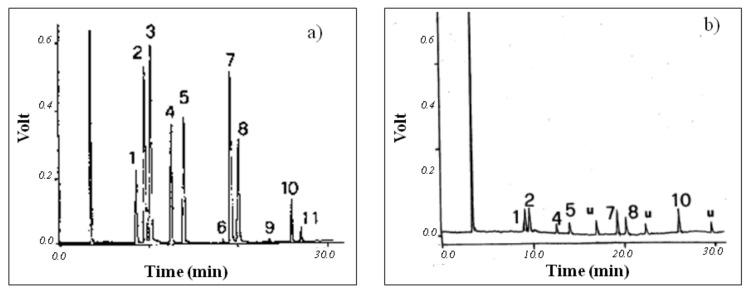
Gas chromatograms of (**a**) a river water sample spiked with a standard solution of halocarbons and (**b**) a real sample (Tiber). For the experimental conditions: see text. Peaks: 1 chloroform (CHCl_3_); 2 hexachloroethane (CCl_3_CCl_3_); 3 tetrachloromethane (CCl_4_); 4 trichloroethene (CCl_2_CHCl); 5 dichlorobromomethane (CHBrCl_2_); 6 trichloroethane (CHCl_2_CH_2_Cl); 7 tetrachloroethene (CHCl_2_CH_2_Cl); 8 dibromochloromethane (CHClBr2); 9 chlorobenzene (C_6_H_5_Cl); 10 bromoform (CHBr_3_); 11 tetrachloroethane (CHCl_2_CHCl_2_); u unknown.

Figure 3 shows the gas chromatograms obtained by headspace analysis of a river water sample spiked with a standard solution of halocarbons (a) and a real sample (Tiber) (b).

Table 3 shows the halocarbon concentrations determined in water samples of the Marta River. First, the values by headspace analysis and liquid–liquid extraction are quite similar, confirming the reliability of the two analytical methods. Looking at the data, the compound concentration does not vary during the sampling period. On the other hand, high concentrations of bromoform, chloroform and tetrachloroethene constitute an interesting result of this research: bromoform levels are probably due to the presence of agricultural crops in the greenhouse, whereas chloroform and tetrachloroethene to the presence of handicrafts installations.

For the Tiber (Table 4), the situation is quite similar: the concentrations do not vary during the sampling period, but the concentrations are lower than those found in the Marta River. This occurrence can easily be explained by the presence of different water purification plants in the Northern Latium territory. The only relevant issue regards a lower presence of tetrachloroethene during the summer period (maybe, due to the closure of activities related to this pollutant).

Finally, a sampling of drinking water performed at different points of Rome’s water supply was carried out to understand the effect of anthropogenic activities on the water quality. This issue is important because the drinking water network is very particular [47]: the water directly “reaches” the Roman population (houses, schools, offices, city standpipes, etc.) from the spring without any chemical-physical pretreatment. Table 5 shows the determination of CHCl_3_, CCl_3_CCl_3_, CCl_4_, CHClCCl_2_, CHBrCl_2_, CHCl_2_CH_2_Cl, CCl_2_CCl_2_, CHBr_2_Cl, C_6_H_5_Cl, CHBr_3_, CHCl_2_CHCl_2_ in different samples.

The results show no significant pollution problems for samples collected at Peschiera-Cittaducale and Peschiera-Castelluccia springs: if the organohalogen compounds were present, their concentration was lower than LOD reported in Table 1. In the other springs and wells (points 2, 3, 5 and 6), significant traces of some organohalogen compounds were found even if the sum is below the law limit (0.87 µg L^−1^, 0.14 µg L^−1^, 1.86 µg L^−1^ and 9.91 µg L^−1^, respectively). This contamination could be related to the different anthropogenic activities [48,49] present in that large area: for instance, halogen compounds are employed in (industrial) laundries, in manufacturing industries of specialty papers and in processes such as degreasing of metal objects, activities largely present in the region.

### 3.2. Inorganic Fraction Characterization

In such kind of studies where the inorganic fraction is deeply investigated by means of nuclear analytical technique (INAA), the analysis of standard reference materials (SRMs) is a fundamental and significant issue [50,51,52]. The sensitivity of the method was evaluated following the definition given by Currie [53] depending on the background level of the interest peak region.

Even if INAA is a well-known and validated analytical methodology, it suffers from interference (Table 2) due to both peak energies overlapping and from fission products. Basically, three interferences are important: two of them could be easily solved by accurate line deconvolution, i.e., the interferences between ^76^As (559.2 keV) and ^122^Sb (564.0 keV) and between ^85^Sr (514 keV) and the annihilation (511 keV); the third interference, a total overlapping between ^75^Se (279.6 keV) and ^203^Hg (279.0 keV), is more difficult and needs the energy calibration curve for evidencing the contribution due to the interfering radionuclide.

Table 6 shows the mean values (expressed as μg g^−1^) along with the standard deviation, the minimum and maximum levels and CV%, for each element determined in the investigated samples. Each value is the average of the element values in all the samples collected in all the areas.

The use of a nuclear technique has allowed a very deep investigation of the investigated samples. More than 40 elements were identified and quantified: among them, interesting levels were found for Hg (up to 6500 μg g^−1^), As (up to 5100 μg g^−1^), Sb (up to 3400 μg g^−1^), Sr (up to 393,000 μg g^−1^), Zn (up to 256,000 μg g^−1^). In contrast, the rare earth elements (REEs), elements also considered to be very important from an economic point of view, showed levels in agreement with the literature data of the crustal composition (Ce 134 μg g^−1^, Dy 3.6 μg g^−1^, Eu 0.97 μg g^−1^, La 55.5 μg g^−1^, Nd 0.76 μg g^−1^, Sm 4.4 μg g^−1^, Tb 1.0 μg g^−1^, Yb 2.5 μg g^−1^) [54]. Further, cadmium is found only in 1 sample in the Bracciano area, whereas it is below the LOD (2 μg g^−1^) in the other samples. According to the element variability, expressed as coefficient of variation (cv%), it is always above 100% except for Ag, Br and Sn and ranges between 101% (Na) and 952% (Zn), showing a large distribution of the elements in the investigated areas. This difference, which describes the large distribution variability of elements in all the areas, will be discussed with different statistical approaches. Another consideration regards one of the targets of such study, i.e., the uranium: it is at 13.6 μg g^−1^ mean level. The mean levels (2–14 μg g^−1^) are quite similar to those present in the Earth’s crust (4 μg g^−1^).58 However, the maximum U levels reached in few samples, i.e., 400 μg g^−1^ (Bracciano), 43 μg g^−1^ (Vulsini area) and 280 μg g^−1^ (Oriolo Romano), permit to consider the site as a sub-economic deposit as previously proposed by other authors [55,56,57].

A very important issue of this study concerns arsenic: it reaches very high concentrations throughout the samples (ranging between 90 μg g^−1^ and 1150 μg g^−1^) in relation to its very low levels in the Earth’s crust (5 μg g^−1^) [58]. This occurrence is very important. One of the most important problems in the Northern Latium region (and particularly in the Viterbo area) is the presence of arsenates in spring and potable waters, which represent a very important issue for such area: periodically, the local authorities are obliged to forbid the use of raw water by the population. Different experiments for avoiding this contamination have been performed during the last decades, but unfortunately, no solutions have been found yet. Further, also Rome is involved in this situation: as reported in a previous paper [59], Rome is characterized by a water network coming directly from springs distant also 100 km far the city. Almost all the water supplies (Marcio, Appio-Alessandrino, Peschiera-Capore, Vergine Nuovo), coming from North and South Rome, “reach” directly the population whereas only one water pipeline, coming from the Bracciano area (Paolo-Traiano pipeline), flows through the Pineta Sacchetti potabilization plant before to be potable.

It should be underlined that the correlation (R^2^) among elements investigated in ores sampled in the Northern Latium locations vs. the Bracciano area is quite good (>0.5), meaning that all the samples show the same provenance and similar average composition [60]. A useful tool for confirming such hypothesis as well as for investigating the natural/anthropogenic element origin comes from the analysis of the enrichment factors (EFs): for each element, the EF (EFx) is calculated according to the following formula:

(1) $${\mathrm{EF}}_{x}=\frac{{\left(\frac{C_{x}}{C_{n}}\right)}_{\mathrm{sample}}}{{\left(\frac{C_{x}}{C_{n}}\right)}_{\mathrm{reference}}}$$

where C_x_ is the element concentrations in the sample and in the reference, i.e., upper continental crust [61], respectively, and C_n_ are the concentration levels of the normalizing element (i.e., Fe or La or Sc) in the sample and in the reference [62], respectively.

The EFs manage to evaluate the soil quality throughout the deficiency/enrichment of the elements. Five classes are identified: EF values < 2, the soil quality show deficiency to minimal enrichment in such elements; EFs ranging between 2 and 5, moderate enrichment; EFs ranging between 5 and 20, significant enrichment; EFs ranging between 20 and 40, very high enrichment; EFs > 40, extremely high enrichment [63,64]. First, the EFs obtained by using the three different normalizing agents (i.e., Fe, La, Sc) show a very similar scheme (Table 7).

By using any normalizing agents, As, Sb, and Hg show the highest EFs, above 80 (i.e., ranging between 298 and 1438 by Fe, 145–699 by La, 82–395 by Sc): it means that anthropogenic sources strongly affect such element values over the geochemical origin. This is particularly important for the assessment of the arsenate content in potable waters, the issue above just stressed. Important elements such as Sr, Zn and Cd show EFs between anthropogenic and natural origins: actually, their EFs are above 40 when Fe is used as a normalizing agent. Th (EFs are 3.07, 1.49 and 0.84 for Fe, La and Sc, respectively) and U (EFs are 7.66, 3.72, 2.10 for Fe, La and Sc, respectively) can be considered no enriched elements. Once again, this is another confirmation that the geochemical hypothesis [56] of considering the Northern Latium area as uranium province/deposit is not justified. Among the REEs, La, Ce, Nd, Sm, Eu, Tb, Dy, Yb, along with Sc were determined: all REEs appear as depleted elements by normalizing agents (EFs < 2), except Ce and Sc using Fe as a normalizing agent.

Further, for a more detailed definition of the soil, the geoaccumulation index (Igeo) has also been studied for few elements, i.e., As, Cd, Cr, Ni, Pb (determination performed by ICP-ES), Zn [16]. This parameter, investigated according to Müller’s definition [38] for metal concentrations in the 2-micron fraction of the soil dust (sampled in Bracciano area), is calculated as follows:

(2) $$\mathrm{Igeo}=\ln\frac{C_{n}}{1.5\times B_{n}}$$

where *C*_n_ is the element concentration level in the soil dust, B_n_ is the geochemical background, and 1.5 is a factor taking into account the possible variations of the background data due to lithological variations [65].

For Igeo parameter, seven classes are identified: Igeo ≤ 0, soil is uncontaminated by that element; 0 < Igeo < 1, soil uncontaminated to moderately contaminated; 1 < Igeo < 2, soil moderately contaminated; 2 < Igeo < 3, soil moderately to heavily contaminated; 3 < Igeo < 4, soil heavily contaminated; 4 < Igeo < 5, soil heavily to extremely contaminated; Igeo ≥ 5, soil extremely contaminated. As geochemical background [66], four different data were considered, such as continental and upper continental crust [59], United States [60] and medium of the world [67]. Hence, in our case, Zn shows Igeo values ranging between 0.69 and 0.99, Cd between −0.72 and 0.38, Ni between −0.62 and 0.48, Cr between −0.84 and 0.45, Pb between 1.35 and 1.60, whereas As confirms its important role in the soil contamination because its Igeo ranges between 1.76 (soil moderately contaminated) and 3.21 (soil heavily contaminated).

**Figure 4:**
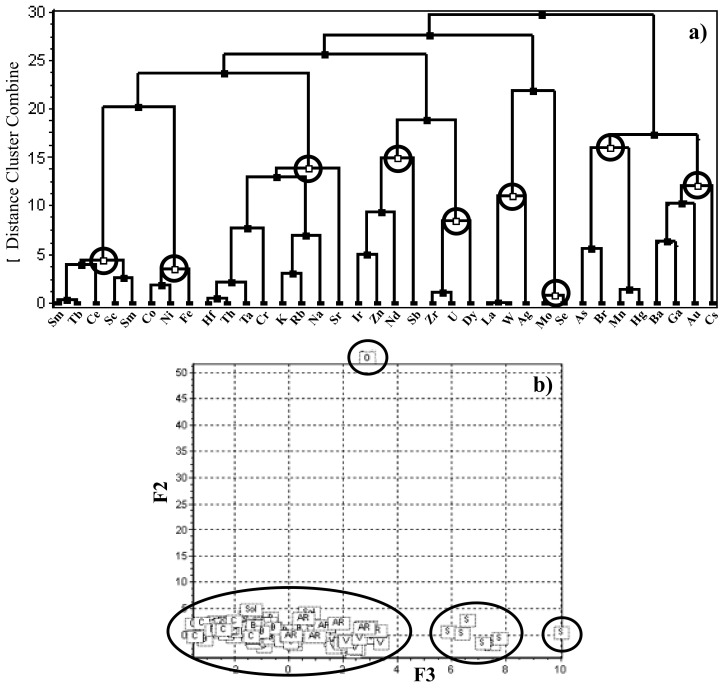
Chemometric approach: (**a**) hierarchical variable clustering analysis based on dendrogram showing the relationship among the elements and (**b**) and principal component analysis (PCA).

Finally, a hierarchical cluster analysis (HAC) study is performed. The correlation among elements is reported in Figure 4a as dendrogram: the elements are grouped in 9 clusters (1st cluster: Hf, Th, Ta, Cr, K, Rb, Na, Sr; 2nd: Co, Ni, Fe; 3rd: Mo, Se; 4th: Eu, Sc, Sm, Ce, Tb; 5th: Dy, U, Zr; 6th: As, Br, Mn, Hg; 7th: La, W, Ag; 8th: Ir, Zn, Nd, Sb; 9th: Ba, Ga, Cs). This analysis was further used for a more specific chemometric analysis [68,69], i.e., the principal component analysis (PCA) (Figure 4b), performed by means of open-source software, Tanagra [70], using the centroid merge method and the Euclidean distance as a measure of proximity [71]. An explorative analysis has considered a data set of the 36 chemical composition variables that, after normalization, allow obtaining 15 principal components describing 81% of the total variance of the data matrix (increasing up to 90.4% with 20 components). The PCA consequent test has evidenced the separation of the samples into three clusters (F2 and F3 were used for a better representation): the principal one is formed by 102 samples, the second one is formed by seven samples collected in Vulsini (signed as S) whereas the only totally different sample comes from Oriolo Romano (as O). It means that the ores have the same origin.

Finally, a simultaneous (and brief) investigation of the atmospheric particulate matter was performed. Airborne particulate matter (PM_10_), fraction believed a risk cause of dangerous effects on human health, including lung cancer, was collected onto a 37 μm filter by means of a Hydra instrument (FAI Instruments, Fonte Nuova, Italy) in the Bracciano area during September 2016. The sampling time was two weeks long: the sample was subjected to the same analytical procedure described previously (irradiation in the nuclear reactor and gamma analysis according to the half-lives of the radionuclides produced), and the data are reported in Table 8 along with literature data in Rome [39]. Both data are very similar except few comparisons such as As, Ba, Cr, Hg, Zn: once again, the arsenic level determined in Northern Latium is higher than the Rome values (almost 3-times fold) even if there are little data for making an assumption on the anthropogenic contributions to the element concentrations.

### 3.3. Effect of Natural/Anthropogenic Risks on the Public Health

One of the major problems in the entire Italian territory is its physical vulnerability, which increases exponentially with the urban and infrastructural expansion to which our cities and the natural physical environment as a whole are subjects [72]. The damages to the territory, unfortunately, are intended to affect public health in terms of increasing diseases or the onset of new pathologies. The large use of anthropogenic sources is undermining the soil and water reservoirs and reducing the availability of the resources for living. In the area investigated in this study, the intensive use of agriculture and the high presence of industrial laundries made a great concern about the halocarbon occurrence. On the other hand, the natural presence of arsenic in the ores contaminated the water resources. In summary, two different kinds of pollution, i.e., natural and anthropogenic pollutants, affect the same area. According to the data determined following the samplings, even if the hazards are present, no risks for public health are evident in the area. Few halocarbons are present at ultra-trace levels (µg L^−1^) whereas as shows good mobility in relation to the seasonal variability. These results do not give any significant worries, but they are indices of an upsetting situation that could change if other different sources arise.

## 4. Conclusions

Anthropogenic activities remain the main cause of poor air quality, but there are also natural sources of air pollution that can play a very important role in this context. Our study shows that this area is worthy of being deeply investigated. Particularly, the study addressed two important issues, i.e., the determination of halogenated volatile organic compounds and heavy metals. The investigated organic compounds are highly volatile compounds that produce negative effects on human health and contribute to the greenhouse effect. Monitoring and limiting the presence of these substances in the atmosphere is thus crucial, in particular where different types of emission sources are present [73]. On the other hand, studies have reported various chronic and subchronic effects from exposure to heavy metals [74]. The area is still contaminated due to the previous presence of a high number of industrial laundries with the consequent high presence of halogenated compounds in the river waters: this occurrence was identified for the first time by such measurements. Further, this study highlights some important issues from toxicological and geological points of view. First, this study shows for the first time that the ores present in the area are responsible for the highest concentrations making the water undrinkable (with the consequence that Italy is under European infringement for that). Second, for a long time, this area has been considered a uranium deposit with high economic relevance, whereas this study demonstrates that this area cannot be considered in this way. These two issues, once again apparently different between them, are important and characterize such entire territory for driving the policy to make a decision for reducing environmental pollution and risks for the public health. In fact, the overall effect of these two sources of pollution can represent, as has now been widely demonstrated, a serious risk to human health. Studies are needed to ensure that public health is better protected. These authors agree with Jacqueline McGlade, formed executive director of the European Environment Agency (EEA) that declared the “poor air quality due to natural sources is, by definition, out of our control, but analyzes have shown that authorities should make further efforts to reduce air pollution as far as they can because the Cumulative effect of man-made and natural particulate matter can seriously harm people’s health”. This statement is really important and evidences the role of natural and anthropogenic sources in environmental studies.

## Figures and Tables

**Table 1:** Linearity range (µg L^−1^), relative standard deviation (RSD, %), recoveries (%) and limit of detection (µg L^−1^) in HS- and LL-GC-ECD analyses for the halocarbons investigated in this work.

Compound	Range	HS Analysis			L–L Extraction Analysis		
		RSD	Recovery	LOD	RSD	Recovery	LOD
	µg L^−1^	%	%	μg L^−1^	%	%	μg L^−1^
CHCl_3_	0.9–12.0	3.0–0.8	98.3	0.040	2.5–0.6	96.2	0.008
CCl_3_CCl_3_	0.8–12.5	3.0–1.0	99.2	0.030	2.6–0.6	95.7	0.006
CCl_4_	0.3–27.2	2.0–1.0	96.4	0.005	2.2–0.5	94.6	0.002
CHClCCl_2_	1.0–11.6	4.0–1.2	99.1	0.020	3.2–0.9	95.6	0.007
CHBrCl_2_	0.9–15.4	3.0–1.1	98.4	0.030	2.3–0.9	93.5	0.008
CHCl_2_CH_2_Cl	0.8–11.0	4.0–1.0	97.3	0.040	3.0–0.6	93.2	0.010
CCl_2_CCl_2_	1.0–14.0	3.0–0.8	94.5	0.010	2.6–0.5	91.6	0.006
CHBr_2_Cl	1.1–16.2	3.0–1.0	98.2	0.060	2.2–0.6	96.1	0.009
C_6_H_5_Cl	2.2–20.5	4.0–0.8	99.1	0.800	2.8–0.7	94.8	0.070
CHBr_3_	1.5–17.8	3.0–0.7	97.8	0.060	2.4–0.6	96.1	0.007
CHCl_2_CHCl_2_	1.1–16.3	4.0–0.8	98.5	0.090	2.8–0.7	95.4	0.008

**Table 2:** Nuclear data and limit of detection (LOD; theoretical in ng, calculated according to ref. [38] in ng g^−1^) of each element determined by nuclear analytical technique (INAA).

Element	Radionuclide	Half-Life ^1^	γ-Ray ^2^	LOD		Interference
			(keV)	Theoretical	Calculated	
Ag	^110m^Ag	250.4 d	657.7	0.1	400	
As	^76^As	26.3 h	559.2	0.001	8	^122^Sb (564.0 keV)
Au	^198^Au	2.70 d	411.8	0.01	1	^152^Eu (411.0 keV)
Ba	^131^Ba	11.5 d	496.3	0.2	10 ^3^	
Br	^82^Br	1.47 d	776.5	0.002	20	^152^Eu (778.6 keV)
Cd	^115^Cd	2.2 d	527.7	0.03	2 ^3^	
Ce	^141^Ce	32.38 d	145.4	0.0001	58	
Co	^60^Co	5.272 y	1332.5	0.06	0.86	
Cr	^51^Cr	27.7 d	320.0	0.1	88	
Cs	^134^Cs	2.062 y	795.7	0.01	1.2	
Dy	^165^Dy	2.35 h	361.7	0.0001	0.01	
Eu	^152^Eu	12.7 y	1408.0	0.0001	0.3	
Fe	^59^Fe	45.1 d	1099.2	20	6.3 ^3^	
Ga	^72^Ga	14.3 h	630.1	0.002	10	^54^Mn (834.8 keV)
Hf	^181^Hf	42.4 d	482.2	0.02	250	
Hg	^203^Hg	46.9 d	279.0	0.04	5.2	^75^Se (279.6 keV)
Ir	^192^Ir	74.3 d	316.5	0.0003	1	
K	^42^ K	12.52 h	1524.7	0.2	260 ^3^	
La	^140^La	40.27 h	1596.2	0.001	3.5	
Mn	^56^Mn	2.6 h	1810.7	0.0008	100	
Mo	^99^Mo	2.75 d	141.0	0.03	1 ^3^	
Na	^24^Na	15.0 h	1368.4	0.004	2.0	
Nd	^147^Nd	11.1 d	531.0	0.0001	1 ^3^	
Ni	^58^Co	70.78 d	810.7	6	80 ^3^	
Rb	^86^Rb	18.66 d	1076.7	0.8	400	
Sb	^124^Sb	60.3 d	1690.7	0.001	6	
Sc	^46^Sc	83.85 d	889.2	0.002	0.9	
Se	^75^Se	120.4 d	264.6	0.1	9	^182^ Ta (264.1 keV)
Sm	^153^Sm	1.948 d	103.1	0.0001	0.41	
Sn	^113^Sn	115.1 d	391.1	9	40 ^3^	
Sr	^85^Sr	64.0 d	514.0	4	50 ^3^	e^+^ +e^−^ (511.0 keV)
Ta	^182^ Ta	115.1 d	1221.2	0.01	200	
Tb	^160^Tb	72.1 d	879.4	0.0001	0.3	
Th	^233^ Pa	27.4 d	311.8	0.01	100	
U	^239^Np	2.35 d	277.6	0.005	30	^203^Hg (279.0 keV) ^76^Se (279.6 keV)
W	^187^ W	24.0 h	685.7	0.001	10	
Yb	^175^Yb	4.21 d	396.1	0.0001	10	
Zn	^65^Zn	243.8 d	1115.5	0.1	12	^46^Sc (1120.1 keV)
Zr	^95^Zr	65.5 d	724.2	1	80 ^3^	

^1^ m minute, h hour, d day; y: years; ^2^ ref. [39]; ^3^ expressed as µg g^−1^.

**Table 3:** Organohalogens in the Marta River samples (n. 24) during different seasons in 2015 and 2016. For each compound are reported the levels determined by the two analytical procedures (headspace analysis (HS)-gas chromatography-electron capture detector (GC-ECD)/liquid–liquid extraction (LL)-GC-ECD).

Compound	Concentration (µg L^−1^)		
	Spring	Summer	Winter
CHCl_3_	3.50/3.21	4.35/4.50	3.60/3.32
CHBrCl_2_	0.20/0.15	0.12/0.10	0.30/0.21
CHBr_2_Cl	0.10/0.11	0.09/0.08	0.10/0.09
CHBr_3_	3.00/2.57	2.39/2.80	3.20/3.00
CCl_3_CCl_3_	1.70/1.65	1.91/1.90	1.10/1.19
CHClCCl_2_	2.60/2.62	3.11/3.00	3.20/3.19
CCl_2_CCl_2_	2.15/2.20	2.30/2.21	2.18/2.25
CHCl_3_	3.25/3.10	4.40/4.32	4.00/3.87
CHBrCl_2_	0.12/0.07	0.09/0.05	0.13/0.10
CHBr_2_Cl	0.06/0.08	0.05/0.04	0.07/0.06
CHBr_3_	3.35/3.20	3.00/2.85	3.20/3.02
CCl_3_CCl_3_	1.20/1.26	1.65/1.83	1.32/1.47
CHClCCl_2_	2.40/2.48	2.92/2.98	3.25/3.35
CCl_2_CCl_2_	2.65/2.75	1.98/2.07	2.32/2.46

**Table 4:** Organohalogens in the Tiber samples (#24) during different seasons in 2015 and 2016. For each compound are reported the levels determined by the two analytical procedures (HS-GC-ECD/LL-GC-ECD).

Compound	Concentration (µg L^−1^)		
	Spring	Summer	Winter
CHCl_3_	2.35/2.15	3.00/2.92	1.96/2.00
CHBrCl_2_	0.08/0.10	n.a./0.06	0.06/0.09
CHBr_2_Cl	n.a./0.11	n.a./0.09	n.a./0.10
CHBr_3_	1.20/1.04	0.81/0.75	1.12/1.05
CCl_3_CCl_3_	1.10/1.20	1.21/1.34	1.30/1.43
CHClCCl_2_	2.00/2.17	1.95/2.10	2.42/2.57
CCl_2_CCl_2_	3.15/3.43	2.00/2.27	3.28/3.41
CHCl_3_	3.00/2.86	2.82/2.85	2.56/2.50
CHBrCl_2_	0.09/0.05	0.05/0.04	0.06/0.05
CHBr_2_Cl	0.05/0.04	0.12/0.08	0.07/0.05
CHBr_3_	1.65/1.82	1.00/1.21	1.56/1.72
CCl_3_CCl_3_	1.21/1.34	1.45/1.56	1.00/1.23
CHClCCl_2_	2.42/2.56	2.00/2.21	2.12/2.34
CCl_2_CCl_2_	3.65/3.76	2.42/2.56	3.45/3.53

n.a.: not analyzed.

**Table 5:** Organohalogen levels (µg L^−1^) determined at different points of Rome’s water supply.

Compound	Sampling Points ^1^					
	1	2	3	4	5	6
CHCl_3_	<0.008	0.45	0.10	<0.008	0.41	1.05
CCl_3_CCl_3_	<0.006	0.12	0.04	0.18	0.07	0.05
CCl_4_	<0.002	0.01	<0.002	0.008	0.01	<0.002
CHClCCl_2_	<0.007	0.13	<0.007	0.10	0.25	<0.007
CHBrCl_2_	<0.008	0.06	<0.008	0.15	0.08	3.03
CHCl_2_CH_2_Cl	<0.010	<0.010	<0.010	<0.010	<0.010	<0.010
CCl_2_CCl_2_	<0.006	0.10	<0.006	0.11	0.06	<0.006
CHBr_2_Cl	<0.009	<0.009	<0.009	0.28	0.36	4.60
C_6_H_5_Cl	<0.070	<0.070	<0.070	<0.070	<0.070	<0.070
CHBr_3_	<0.007	<0.007	<0.007	0.49	0.72	1.18
CHCl_2_CHCl_2_	<0.008	<0.008	<0.008	<0.008	<0.008	<0.008

^1^ sampling points: 1 Peschiera-Cittaducale spring; 2 Rifolta and Torre Angela wells; 3 Marcio aqueduct; 4 Peschiera-Castelluccia spring; 5 Torre Nova water center; 6 San Polo Marcio aqueduct.

**Table 6:** Mean values (μg g^−1^ ± standard deviation (SD)), variability (as coefficient of variation, cv%, data reported in brackets) and min–max values for each element determined in overall the samples investigated.

Element	Mean ± SD (cv%)	Min–Max
Ag	0.10 ± 0.04 (36.6)	1–5100
As	414 ± 915 (220.8)	1–5100
Au	0.007 ± 0.010 (141.2)	10–268,000
Ba	7838 ± 30,912 (394.4)	1.0–40.0
Br	6.63 ± 5.28 (79.7)	<0.020–12
Ce	134 ± 191 (143.1)	0.7–980
Co	29.8 ± 105.5 (354.0)	0.07–790
Cr	39.0 ± 48.3 (123.9)	0.4–325
Cs	25.9 ± 47.6 (183.4)	0.05–310
Dy	3.62 ± 5.88 (162.4)	0.05–45
Eu	0.97 ± 1.33 (137.2)	0.01–8.3
Fe	47,813 ± 85,180 (178.2)	180–410,000
Ga	12.6 ± 18.1 (143.6)	0.8–126
Hf	5.88 ± 6.46 (109.8)	0.02–21
Hg	88.7 ± 662.1 (745.6)	0.01–6500
Ir	0.006 ± 0.011 (173.1)	0.001–0.1
K	13,508 ± 18,896 (139.9)	100–85,000
La	55.5 ± 115.6 (208.2)	0.3–780
Mn	4960 ± 16,992 (342.6)	3.0–131,000
Mo	14.3 ± 26.1 (182.8)	3.0–250
Na	3210 ± 6478 (101.8)	30–47,000
Nd	0.76 ± 1.08 (142.6)	0.05–7.0
Ni	29.0 ± 74.9 (258.7)	0.7–700
Rb	82.3 ± 125.1 (152.0)	1–680
Sb	77.7 ± 372.3 (478.8)	0.1–3400
Sc	7.0 ± 10.5 (149.0)	0.1–91
Se	0.35 ± 0.86 (245.7)	0.004–8.0
Sm	4.4 ± 6.7 (152.9)	0.08–38
Sn	21.4 ± 16.8 (78.6)	4–80
Sr	12,039 ± 55,741 (463.0)	30–393,000
Ta	0.58 ± 0.66 (113.2)	0.005–3.2
Tb	1.04 ± 1.67 (160.1)	0.04–9.0
Th	19.2 ± 23.2 (120.7)	0.06–80
U	13.6 ± 48.0 (353.2)	0.1–400
W	26.5 ± 62.5 (235.8)	1.0–360
Yb	2.52 ± 4.53 (179.7)	0.02–28
Zn	2565 ± 24,429 (952.6)	1–256,000
Zr	103 ± 122 (118.6)	4–1200

**Table 7:** Synoptic table of the calculated enrichment factors (EFs) (ordered in ascending order) for overall the elements (Fe, La, Sc normalizing agents).

Element	Fe	La	Sc
Nd	0.02	0.01	0.01
Na	0.18	0.09	0.05
Ni	0.38	0.18	0.10
Cr	0.41	0.20	0.11
Ta	0.44	0.22	0.12
Eu	0.60	0.29	0.16
Sm	0.81	0.40	0.22
Zr	0.83	0.40	0.23
Ga	0.86	0.42	0.24
Dy	0.90	0.44	0.25
K	0.95	0.46	0.26
Fe	1.00	0.49	0.27
Yb	1.05	0.51	0.29
Tb	1.13	0.55	0.31
In	1.15	0.56	0.32
Co	1.33	0.65	0.37
Rb	1.37	0.66	0.38
Ag	1.59	0.77	0.44
La	2.06	1.00	0.57
Au	2.34	1.14	0.64
Ce	2.63	1.28	0.72
Hf	2.72	1.32	0.75
Th	3.07	1.49	0.84
Cl	3.27	1.59	0.90
Br	3.44	1.67	0.94
Sc	3.64	1.77	1.00
Mn	6.07	2.95	1.67
U	7.66	3.72	2.10
Ir	8.24	4.01	2.26
Se	9.10	4.42	2.50
I	11.20	5.44	3.08
Cs	12.93	6.29	3.55
Sn	13.19	6.41	3.62
Mo	15.43	7.50	4.24
Ba	26.06	12.67	7.16
W	28.65	13.92	7.87
Sr	40.65	19.76	11.17
Zn	43.76	21.26	12.02
Cd	82.74	40.21	22.72
As	298.56	145.09	82.00
Sb	503.46	244.67	138.28
Hg	1438.28	698.97	395.02

**Table 8:** Element concentration level (ng m^−3^) in PM_10_ filter sampled during the study and compared with Rome’s levels reported in [48].

Element	Bracciano	Rome
Ag	0.194	0.176
As	3.59	1.35
Au	0.163	0.008
Ba	55.8	12.8
Br	39.1	22.2
Cd	0.892	0.526
Ce	0.571	0.843
Co	0.236	0.379
Cr	10.4	7.28
Cs	0.127	0.151
Dy	0.028	n.d.
Eu	0.015	0.012
Fe	488	566
Hf	0.089	0.020
Hg	0.283	1.07
K	2400	1100
La	0.403	0.188
Mn	59.8	40.0
Mo	4.15	2.10
Na	1890	420
Nd	0.311	0.245
Ni	5.13	4.54
Rb	0.981	2.19
Sb	12.4	9.22
Sc	0.021	0.046
Se	0.794	0.687
Sm	0.112	0.053
Sr	33.5	n.d.
Th	0.453	0.204
U	3.65	n.d.
W	0.316	1.25
Yb	0.023	0.015
Zn	54.2	80.0
Zr	0.194	n.a.

n.a.: not analyzed; n.d.: not detected.

## Data Availability

Data is available upon request by contacting the corresponding author.
